# Design of PM2.5 monitoring and forecasting system for opencast coal mine road based on internet of things and ARIMA Mode

**DOI:** 10.1371/journal.pone.0267440

**Published:** 2022-05-05

**Authors:** Meng Wang, Qiaofeng Zhang, Caiwang Tai, Jiazhen Li, Zongwei Yang, Kejun Shen, Chengbin Guo

**Affiliations:** 1 College of Mining Engineering, Liaoning Technical University, Fuxin, China; 2 Shenzhen Mixlinker Networks Co., Ltd., Shenzhen, China; Norfolk State University, UNITED STATES

## Abstract

The dust produced by transportation roads is the primary source of PM2.5 pollution in opencast coal mines. However, China’s opencast coal mines lack an efficient and straightforward construction scheme of monitoring and management systems and a short-term prediction model to support dust control. In this study, by establishing a PM2.5 and other real-time environmental information to monitor, manage, visualize and predict the Internet of things monitoring and prediction system to solve these problems. This study solves these problems by establishing an Internet of things monitoring and prediction system, which can monitor PM2.5 and other real-time environmental information for monitoring, management, visualization, and prediction. We use Lua language to write interface protocol code in the APRUS adapter, which can simplify the construction of environmental monitoring system. The Internet of things platform has a custom visualization scheme, which is convenient for managers without programming experience to manage sensors and real-time data. We analyze real-time data using a time series model in Python, and RMSE and MAPE evaluate cross-validation results. The evaluation results show that the average RMSE of the ARIMA (4,1,0) and Double Exponential Smoothing models are 12.68 and 8.34, respectively. Both models have good generalization ability. The average MAPE of the fitting results are 10.5% and 1.7%, respectively, and the relative error is small. Because the ARIMA model has a more flexible prediction range and strong expansibility, and ARIMA model shows good adaptability in cross-validation, the ARIMA model is more suitable as the short-term prediction model of the prediction system. The prediction system can continuously predict PM2.5 dust through the ARIMA model. The monitoring and prediction system is very suitable for managers of opencast coal mines to prevent and control road dust.

## 1. Introduction

There is little research on China’s PM2.5 dust concentration monitoring of opencast coal mine roads. According to Article 642 in the Coal Mine Safety Regulations [1], the total dust concentration of opencast coal mines is measured once a month, and respiratory dust concentration is measured once a month. Because of the lack of real-time monitoring data, coal mine dust pollution in opencast coal mines can only be qualitatively evaluated without quantitative analysis [2].

In order to study the numerical simulation of PM10 diffusion in an opencast coal mine, scholars such as Chinthala Sumanth and Mukesh Khare [3] need to collect PM10 data from multiple stations in multiple periods and finally get a method for estimating PM10 pit retention. Tang Wanjun, Cai Qingxiang [4], and other scholars use β Ray particle monitor and laser monitor to collect data in the opencast coal mine, and they simulate the dust distribution in the opencast coal mine based on fluent. Finally, they get the relationship function between PM2.5 and PM10 and the spatial motion law of dust. Qingyu Guan, Fuchun Li [5], and other scholars downloaded environmental data such as PM2.5 concentration from the urban air quality real-time publishing platform of the China National Environmental Monitoring Center; particulate matter’s temporal and spatial behavior (PM) in Lanzhou is revealed through backward trajectory analysis.

Monitoring equipment is required to collect data, but different sensor devices have different standard specifications and interfaces [6]. Therefore, microcontrollers usually control sensor nodes [7, 8]. Linux [8], Raspberry Pi [9], and C language [10] are often used to build the operating system of sensors. Users need to use different configuration codes according to the actual situation. In order to quickly obtain the information of particulate matter (PM) concentration, Marek Badura, Piotr Batog [9], and other scholars started from the equipment layer [11], connected with the sensor through the microcontroller, and then corrected the sensor accuracy and sensor deviation [12], and successfully built a sensing layer node with reasonable accuracy.

Currently, building data acquisition nodes according to the needs are the mainstream direction. Table 1 summarizes the characteristics of current dust data acquisition.

**Table 1:** Status of dust data acquisition.

Data acquisition method	Characteristic
Manual collection	High consumption of human and material resources
Instrument acquisition	Low degree of visualization and cumbersome data management
Download from a third-party platform	High dependence and limited monitoring location
Building sensor nodes	Low degree of systematization and difficult to expand

As can be seen from the above example, their method of obtaining the original data is very cumbersome, and the learning cost of establishing the Internet of things monitoring system from scratch is too high, so there is no good road PM2.5 monitoring scheme in the opencast coal mine so far. Therefore, to facilitate the acquisition of research data and help the managers of opencast coal mines simplify the construction of monitoring nodes, we use the technology of the Internet of things platform to build monitoring nodes.

Internet of things platform is the application and practice of Internet of things technology. Users can quickly build the Internet of things monitoring system through the Internet of things platform [13] and then collect and analyze data. Analyzing data aims to realize Digital Twin (DT) in the Internet of Things platform, which means that the information exchange between the physical and information layers could be bidirectional [6]. Classification regression analysis [14], neural network [15], time series, and other analysis methods are often used in the prediction work of the service layer, and the use of time series analysis PM2.5 in the service layer has been proved to be feasible [16]

The single Exponential Smoothing method is suitable for analyzing time series without trend. In order to better predict time series with trend components, the Double Exponential Smoothing method (DES) can be used. DES model is one of the methods to analyze time series [17] and can also make a short-term prediction of power load [18]. Moreover, the DES model is mainly used for short-term prediction [19].

The Autoregressive Integrated Moving Average model (ARIMA) is also a statistical model to analyze and predict time series. ARIMA has achieved good prediction results in predicting energy consumption and greenhouse gas emissions [20]. When ARIMA predicts climate variables, the prediction results agree with the data trend [21].

Their research is based on data sets that will not change. However, in practice, the concentration of road PM2.5 changes. In order to make the time series model more suitable for real-time data, we choose real-time monitoring data to build prediction models and prediction systems.

The dust generated by the transportation road is the primary source of PM2.5 pollution in an opencast coal mine [20]. In order to monitor the change of PM2.5 in opencast coal mine roads in real-time and predict the change of dust concentration, and also help managers quickly build the required sensing layer nodes, this study proposes a system based on the Internet of things platform to collect PM2.5 dust data and analyze the data.

We require that the monitoring and prediction system monitor the change of road environmental information of opencast coal mines in real-time. The system has the functions of overrun warning, visualization, and data analysis.

The main contributions of this paper are as follows:

1. We have built a dust information monitoring and management system using Internet of things technology, which solves the problems of complex environmental data collection and cumbersome management. Connecting the sensor with the APRUS adapter replaces the traditional scheme of connecting the sensor with a microcontroller. The general standard specification and interface protocol simplify the construction process of sensing layer nodes. At the same time, the customized visualization function of the Internet of things platform facilitates the use of managers without programming experience. We provide the design process, prototype, and visualization effect for the construction of the monitoring system.
2. This paper analyzes the conversion process between PM2.5 real-time monitoring data and time series. Through cross-validation (CV) [22], we compared the modeling performance of the DES model and ARIMA model in the real-time data of PM2.5 and proved that the DES model and ARIMA model have good feasibility and accuracy in the short-term prediction of PM2.5. We selected the best short-term prediction model for the road PM2.5 prediction system of an opencast coal mine through discussion.

The rest of this paper is organized as follows: the second part introduces the structure construction process of the Internet of things monitoring system, including introducing the APRUS adapter and the use effect display of the Internet of things platform. In Section 3, we conducted data preprocessing, modeling, and cross-validation. In Section 4, according to the evaluation index, the model’s characteristics, accuracy, feasibility, and applicability are discussed, and we choose a suitable short-term prediction model. The last part is the conclusion of this paper.

## 2. Monitoring system

### 2.1 IoT platform

In 1999, Professor Kevin Ashton first proposed the concept of IoT (Internet of things) [11]. The 2010 work report of the Chinese government [23] also made requirements for the development of the Internet of things.

**Fig 1:**
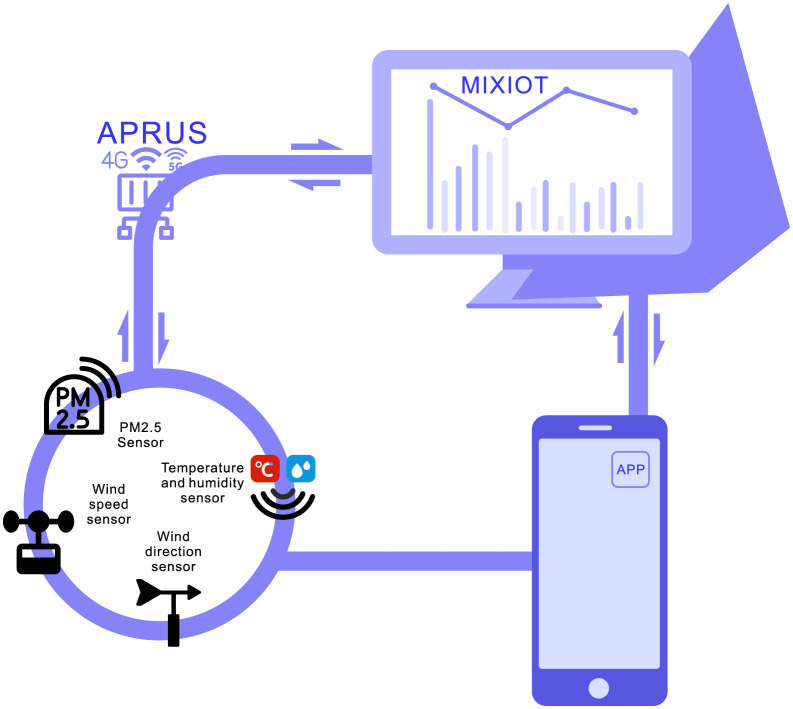
Schematic diagram of PM2.5 monitoring system.

Shenzhen smart IOT Network Co., Ltd., founded in 2014, is one of China’s earliest industrial Internet solution providers. It has developed the MIXIOT IoT platform. The schematic diagram of the monitoring system proposed in this paper based on the Internet of things technology is as follows (Fig 1).

### 2.2 Hardware device

PM2.5 and PM10 dust concentration sensors, temperature and humidity sensors, wind speed sensors, and wind direction sensors are selected in this study. The specific parameters of the sensors are shown in (Fig 9).

**Fig 2:**
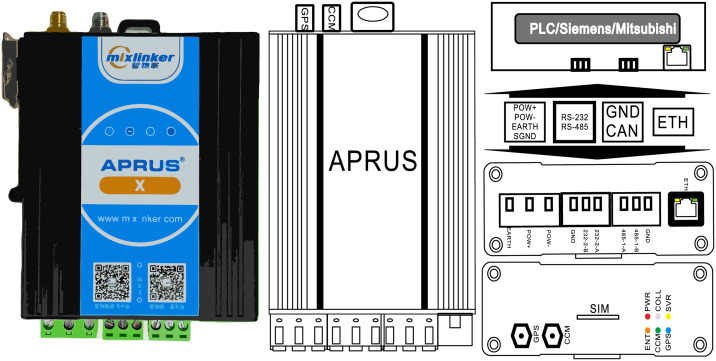
APRUS adapter.

APRUS (Advanced Programmable Remote Utility Server) is an adapter produced by Shenzhen smart IOT Network Co., Ltd. It uses Lua language [24] to write the sensor’s data upload rules and communication interface protocol. The interface and appearance of the adapter are shown in the figure (Fig 2). The adapter has interfaces such as RS232, RS485 [25], CAN [26], and Siemens PLC [27]. The adapter can also report and log sensor faults [28] or send instructions to the equipment to modify or control parameters. Therefore, APRUS can connect most industrial control systems.

### 2.3 Connection

**Fig 3:**
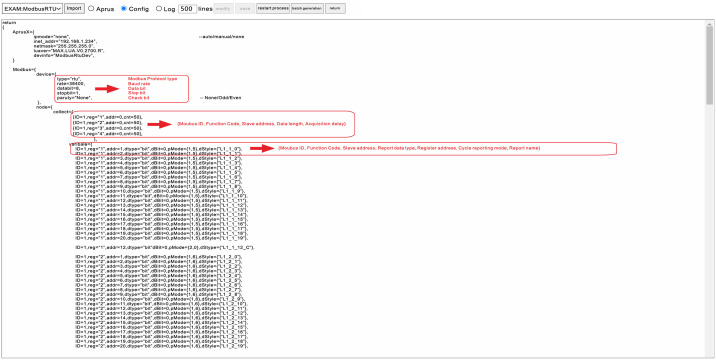
Data interaction rule code.

(Fig 3) shows part of the code of the data acquisition rules of the sensor. The manager needs to create the parameter object according to the format of the proposed frame [29]. Modbus protocol is adopted this time, so we only annotate Modbus-RTU [25].

**Fig 4:**
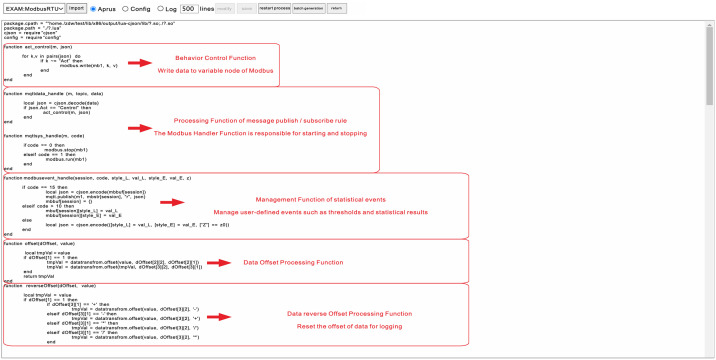
Protocol adaptation code.

The parameters to be transferred in the interface attribute of the parameter object include: baud rate, data bit, stop bit, and check bit of the sensor interface. The objects of the acquisition node include Modbus ID, address, function code, data length, and interval time (ms). For each sensor node, the objects of the reporting node include Modbus ID, function code, slave address, reporting data type, register address, reporting cycle, and reporting data label. The MQTT [30] processing function (Fig 4) can import the parameter object. The Internet of things server subscribes to the theme of each node according to the parameter object and then stores it in different databases according to the label and separator. Sensors with different protocols and communication interfaces can only be connected to the Internet of things platform by parameter objects.

**Fig 5:**
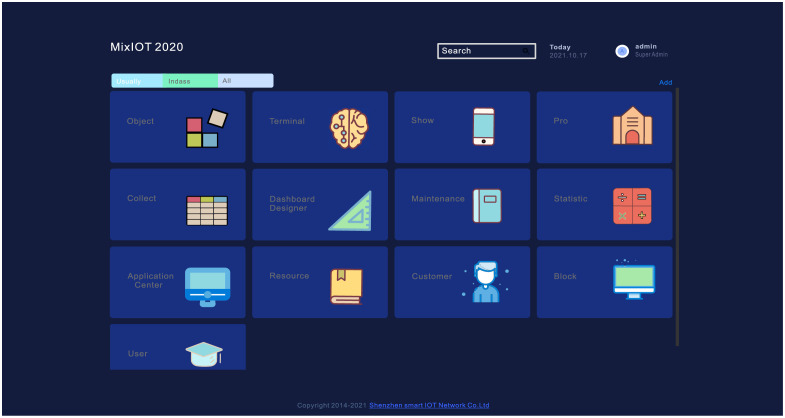
MIXIOT platform.

The sensor is set to collect and upload data every 5 seconds, and the data is uploaded to the MIXIOT platform (Fig 5). The manager can know the pollution degree of road PM2.5 in real-time by viewing the monitoring data. When the PM2.5 concentration at the monitoring point exceeds the set threshold, the MIXIOT platform will alarm and remind the administrator through the web page or intelligent mobile device application.

**Fig 6:**
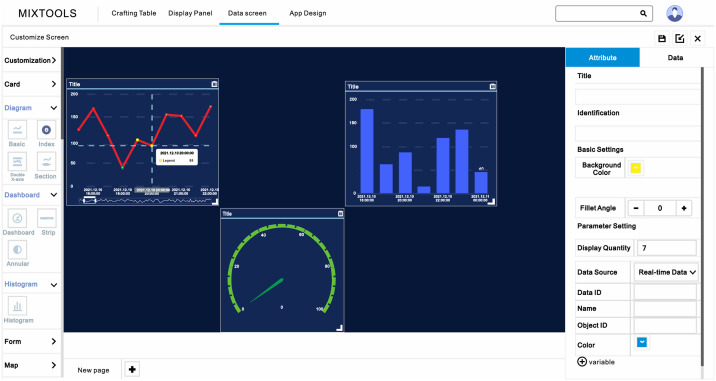
Customize visualization scheme.

**Fig 7:**
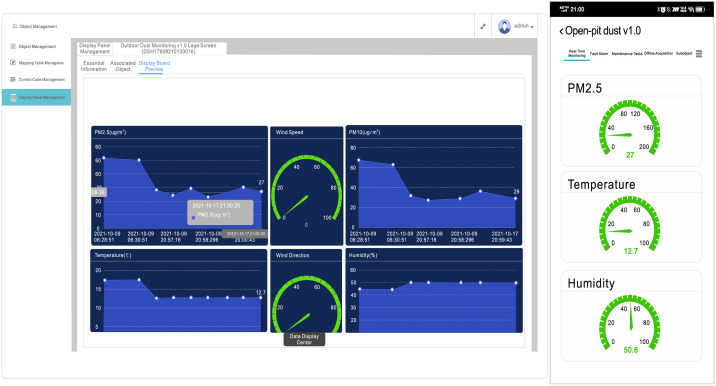
Visualization of monitoring data.

The IoT system usually uses Front End Programming Languages (JavaScript, HTML, CSS) [31–33] to visualize data, but the IoT platform can customize the visualization interface. The Internet of things platform modularizes the graph, dashboard, and other components, and the components and data are connected through the reporting name in the data interaction rule code. As shown in (Figs 6 and 7), the manager can build the website interface and the visualization interface of an intelligent mobile device application by selecting the visualization method, the source of data, and adjusting the location and size.

**Fig 8:**
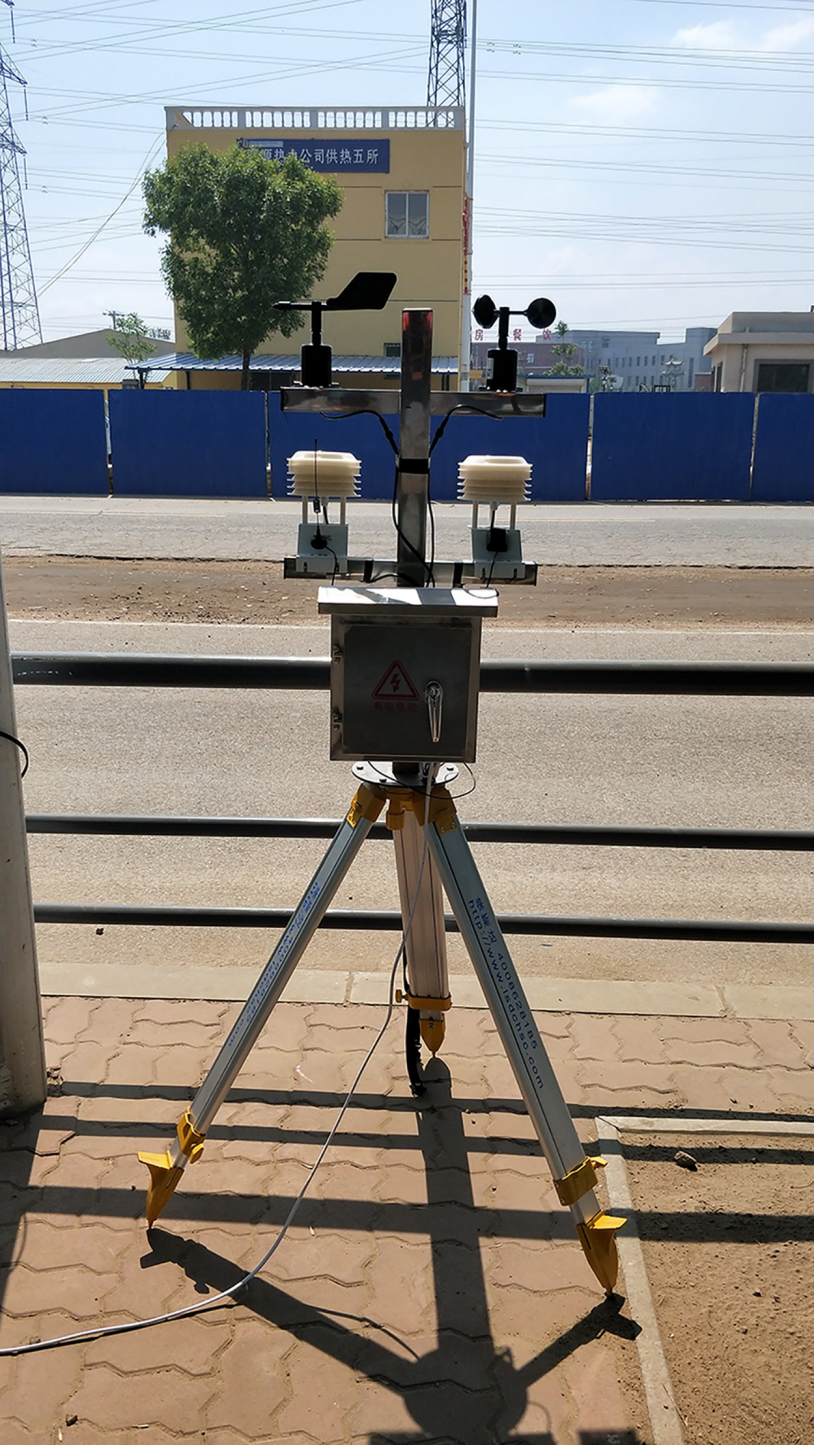
Monitoring device.

**Fig 9:**
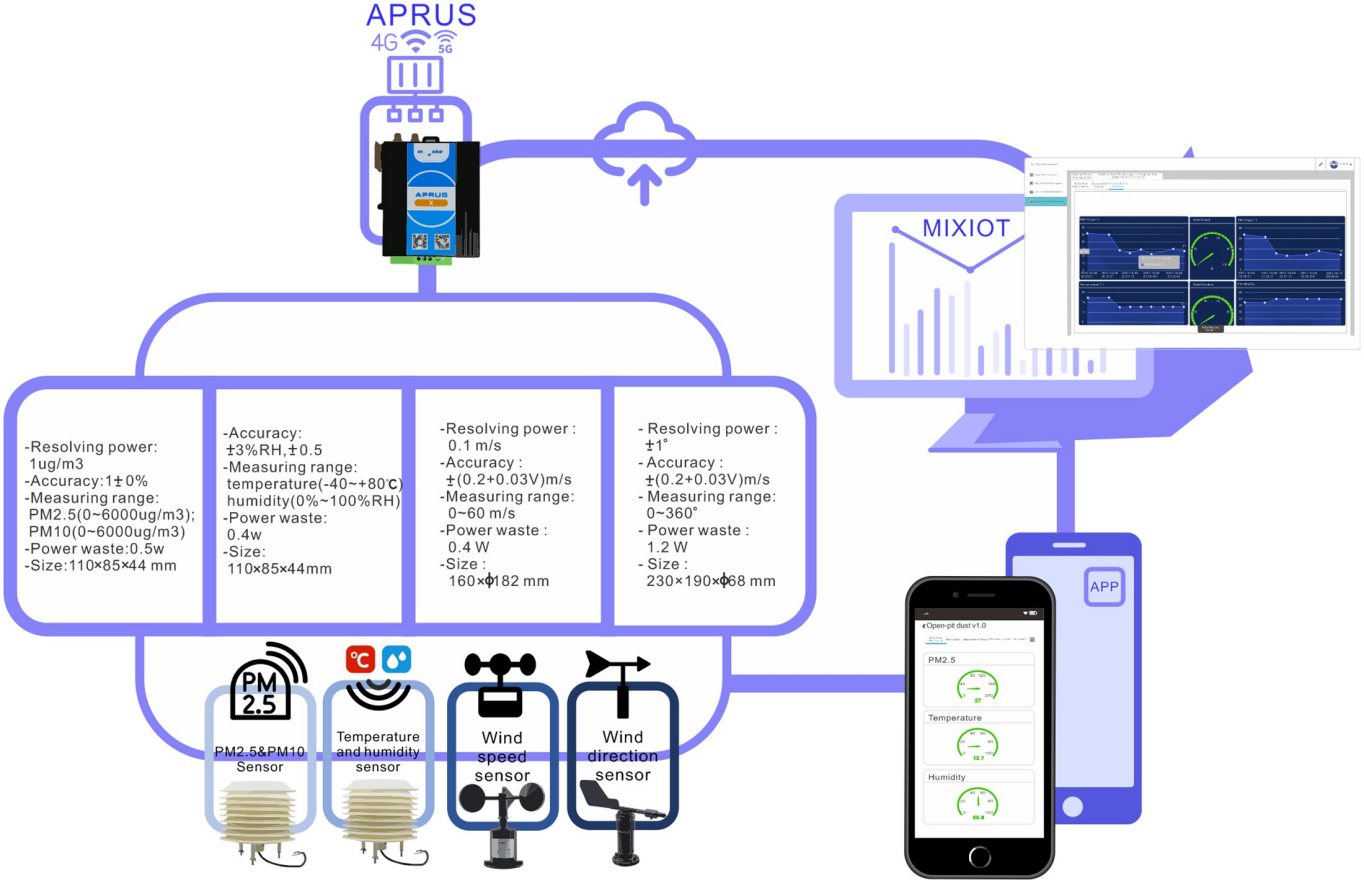
Overall architecture.

PM2.5 monitoring equipment is assembled by support (Fig 8). (Fig 9) shows the overall architecture of the Internet of things opencast coal mine road PM2.5 monitoring system.

## 3. Construction of prediction model

### 3.1. Data sources

**Fig 10:**
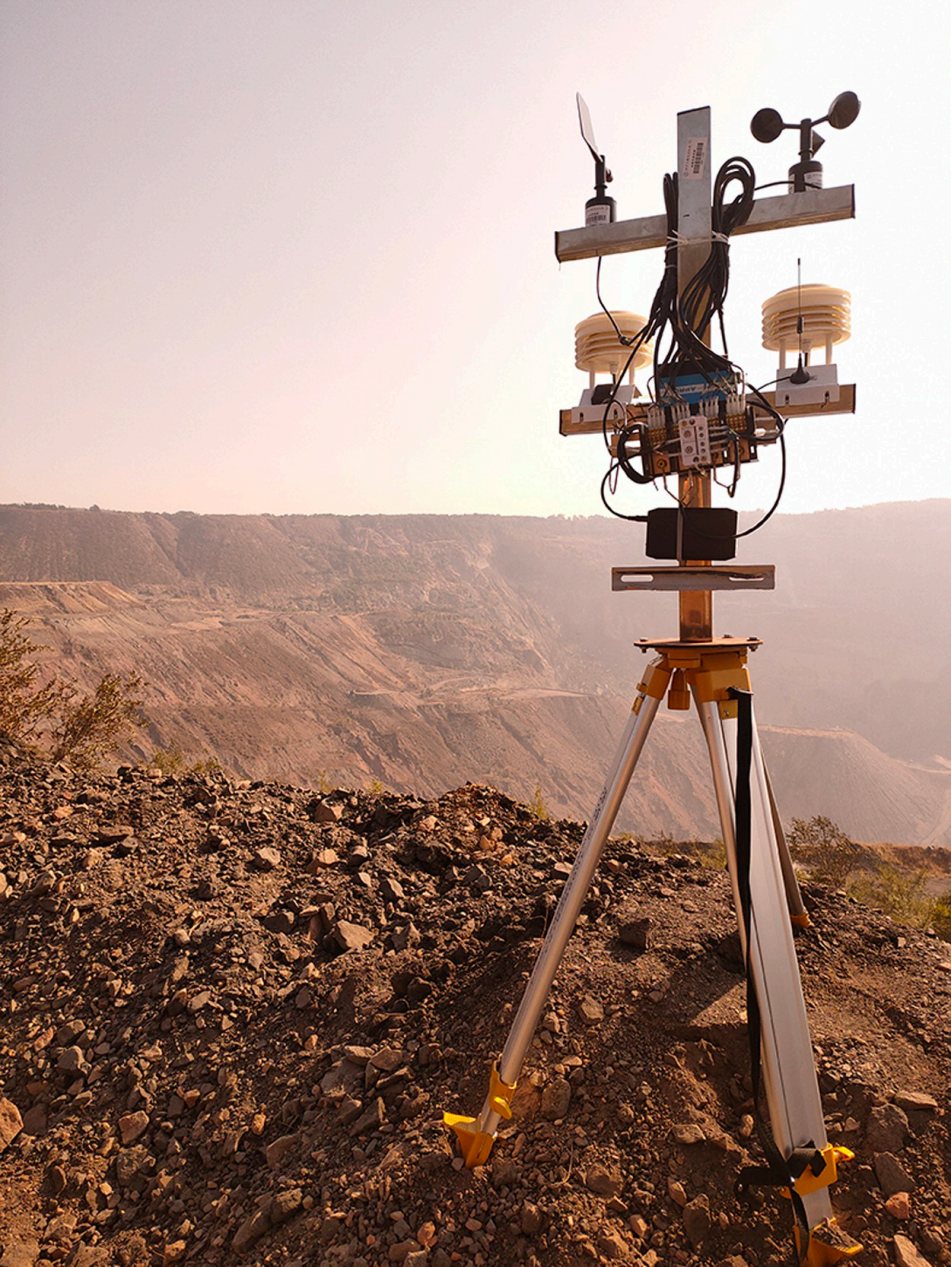
No. 5 monitoring point of transportation road of opencast coal mine.

Install the monitoring device at monitoring point 5 of the transportation road of the opencast coal mine (Fig 10).

### 3.2 Strategies and methods

We set the data upload rules of the sensor online through the APRUS adapter, and the data is transmitted once every 5 seconds. Considering the network delay, we will preprocess the data, and the goal is to process the data into a standard time series [34]. After decomposing the time series [35], we will obtain the trend, season, and residual.

DES model is characterized by considering the weighted average and change trend of historical data, and the ARIMA model also considers the weight and trend of historical values. Interestingly, using a small number of samples for short-term prediction also has good accuracy [36]. The length of data we will choose this time is 661 (one hour). The preprocessed data is divided into the training set and test set. We conduct processing and parameter estimation on the training set and model evaluation on the test set.

DES model is suitable for time series with time trends [18]. We construct the objective function according to the constraint range of smoothing parameters and the model accuracy evaluation standard MAPE. After solving the local optimal smoothing parameters, we will fit the model, cross verify, and predict.

ARIMA model has high requirements for the stationarity of time series. Therefore, we test the stationarity of time series through differencing [21] and ACF (autocorrelation function) [35] and then use ADF (augmented Dickey fuller test) [37] to judge the stationarity further. If the time series is unstable, the time series trend is weakened by differencing. Finally, we perform parameter estimation and cross-validation of the model.

It is worth noting that most researchers use ACF and PACF (partial autocorrelation function) to estimate the parameters of the ARIMA model [21, 36, 38]. In order to avoid the influence of subjectivity in parameter estimation, we will use more objective AIC (Akaike information criterion) and BIC (Bayesian information criterion) evaluation criteria [39] for parameter estimation. We will select the optimal parameters according to the differencing times, AIC and BIC.

In order to evaluate the performance of the model, both the DES model and ARIMA model use generalized cross-validation on the test set [22]. In order to confirm whether the selected model belongs to the best model, the ARIMA model will be subject to residual analysis and the Ljung-Box test [40]

### 3.3 Time series cross-validation

Using cross-validation or Monte Carlo [41] can effectively estimate the model’s performance. However, the time series does have a time structure. If this structure is not retained, the values cannot be randomly mixed in the folding otherwise, all the time dependencies between the observations will be lost [42].

**Fig 11:**
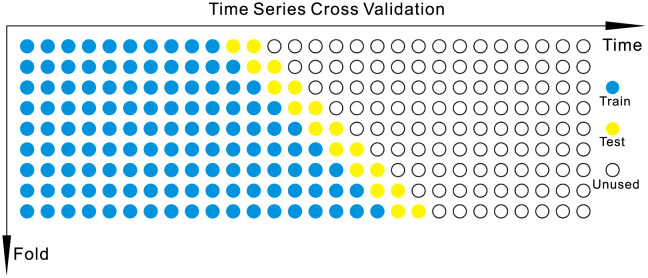
Time series cross-validation.

Richard Morton, Emily L. Kang compared the cross-validation methods of time series such as the P method [22], GCV, LCV and VCV [43]. P method is the most stable compared with other methods. P method is the Preliminary method, and the Preliminary method is similar to the Out-Of-Sample (OOS) approvals [42]. The following figure (Fig 11) shows the Preliminary method of cross-validation of time series.

### 3.4 Data acquisition and preprocessing

There are many ways and objectives to analyze data. In addition to using the third-party data analysis platform [44], Python’s ARIMA model analysis tool can also be used [45].

**Fig 12:**
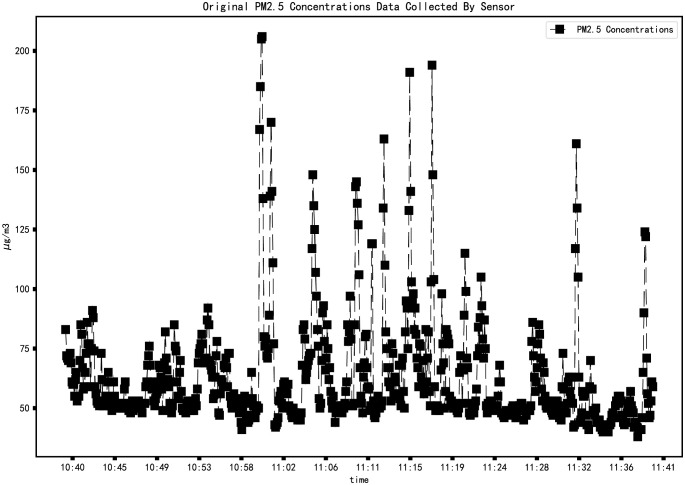
PM2.5 concentration change from 10:40 to 11:40 on October 18, 2021.

We use Python to access the MIXIOT API (Application Programming Interface) to obtain data. The request library in Python is used to obtain the PM2.5 concentration data of the opencast coal mine monitoring site. The time interval is from 10:40 on October 18, 2021, to 11:40 on October 18, 2021, with 661 data pieces, as shown in (Fig 12).

**Fig 13:**
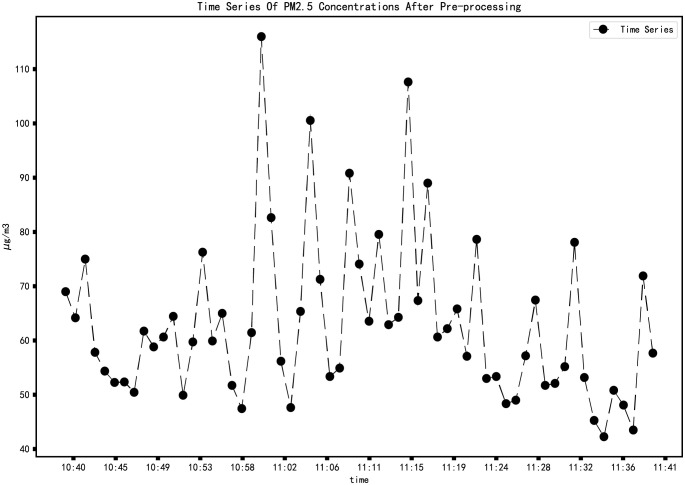
Time series of PM2.5 concentrations.

The upload cycle of the sensor is 5 seconds. Due to the existence of delay, the time interval of the original data is not equidistant, and the original data does not belong to the standard time series [34]. A small amount of sample data can be used when the time series model is used for short-term prediction [36]. So, take the average of the data in one minute to represent the PM2.5 concentration in that minute and regenerate the time series. The time label of the data is from 10:40 on October 18, 2021, to 11:40 on October 18, 2021, with a total of 61 data. The pre-processed data are shown in (Fig 13).

**Fig 14:**
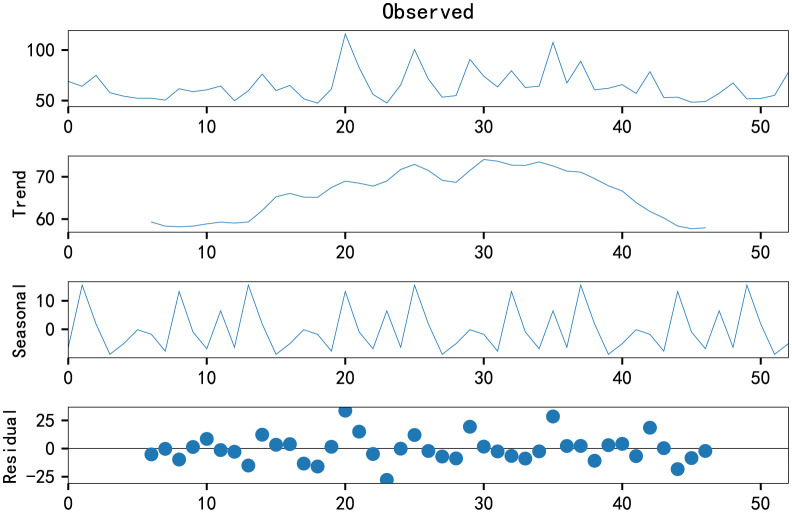
PM2.5 time series decomposition.

To understand PM2.5 characteristics of time series, we decompose the time series, as shown in (Fig 14). We can get the trend, season, and residual of the time series.

When the opencast coal mine is in the peak period of transportation operation, we conducted data collection. It can be seen from (Fig 12) that although the PM2.5 concentration fluctuates wildly, there is a trend before the concentration reaches the peak. The standard time series of the original data (Fig 13) proves this.

We can see from the decomposition diagram (Fig 14) that the first half and the second half of the trend are different. DES model is suitable for time series with the trend, while the ARIMA model is more suitable for stationary time series. Therefore, the DES model can be fitted only by using standard time series, and the ARIMA model needs to be further discussed.

### 3.5 Double exponential smoothing model

Single exponential smoothing and double exponential smoothing can analyze time series, but the use environment of these two methods is different. The single exponential smoothing equation is as follows:

$${\hat{y}}_{t}=\alpha ∙x_{t}+(1-\alpha )∙x_{t-1}$$ (1)

Single exponential smoothing only makes a weighted average of *t* pieces of data in history without considering the trend factor of time series. Therefore, double exponential smoothing needs to be further used. The equation is as follows:

$$l_{t}=\alpha x_{t}+(1-\alpha )(l_{t-1}+b_{x-1})\;0\leq \alpha \leq 1$$ (2)

$$b_{t}=\beta (l_{t}-l_{t-1})+(1-\beta )b_{x-1}\;0\leq \beta \leq 1$$ (3)

$${\hat{y}}_{t+1}=l_{t}+b_{t}$$ (4)

$$l_{0}=x_{0}$$ (5)

$$b_{0}=x_{1}-l_{0}$$ (6)

In the equation, *l*_*t*_ stands for intercept, *b*_*t*_ stands for trend, *α*, *β* Represents the smoothing coefficient. It can be seen from the equation that the predicted value depends on the intercept term *l*_*t*_ and trend item *b*_*t*_. Intercept and trend terms depend on smoothing parameters *α* and *β*

Eqs (2), (3) and (4) are combined to obtain the following equation:

$$Z=\frac{1}{n}\sum_{t=1}^{n}|\frac{\hat{l}+b_{t}-l_{t}}{l_{t}}|\times 100\%$$ (7)

In order to get the optimal local solution, we import the scipy.optimize package in Python. Combined with boundary constraints, the objective function Eq (7), and the least square method, we can get the optimal local solution of *α*, *β*. The iteration results are shown in the following Table 2:

**Table 2:** Iterative results.

MAPE	*α*, *β*	Iterations
5.72%	0.9,0.02	173

**Fig 15:**
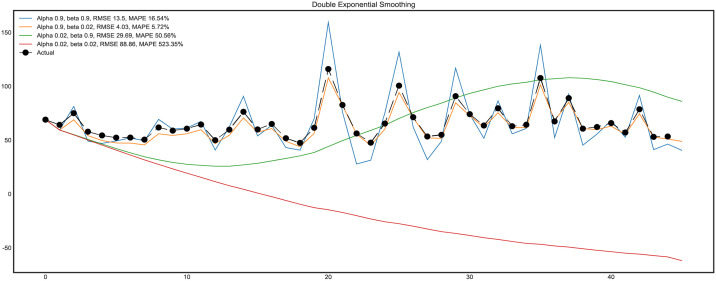
Fitting results of DES model with different parameters.

The fitting results are shown in (Fig 15). It can be seen from the figure that RMSE is 4.03 and MAPE is 5.72%. DES model has high fitting accuracy. The smoothing parameters are α and β; The former is responsible for smoothing the sequence around the trend, while the latter is responsible for smoothing the trend itself. The larger the value, the greater the weight of the latest observation value and the lower the smoothness of the model sequence.

**Fig 16:**
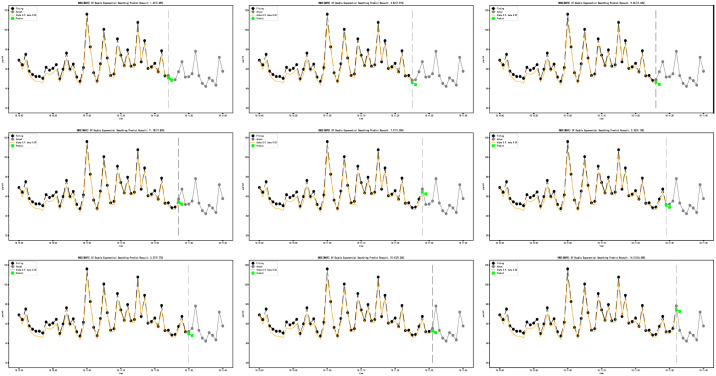
Cross-validation of DES model.

Input the training data and smoothing parameters into the model for cross-validation (Fig 16). The prediction range is one minute ahead of the current sample. In the cross-validation process, the average MAPE is 12.9%, proving that the DES model has good prediction performance for the short-term prediction of PM2.5. However, the prediction range of the DES model is limited. In order to improve this situation, we use the ARIMA model. Moreover, the further analysis still needs to consider the trend factors of time series.

### 3.6 ARIMA model

The basic idea of the time series prediction method is to use the past behavior of data to predict the future trend and change [34]. ARMA (Autoregressive Moving Average) is a time series analysis model proposed by American statistician box and British statistician Jenkins in 1976. It is the most commonly used model for fitting stationary series [46]. The form of ARMA (p,q) model can be described as:

$$x_{t}=\phi_{1}x_{t-1}+\phi_{2}x_{t-2}+\cdots +\phi_{p}x_{t-p}+\varepsilon_{t}-\theta_{1}\varepsilon_{t-1}-\cdots -\theta_{q}\varepsilon_{t-q}$$ (8)

In Eq (8), *ϕ*_1_, ⋯, *ϕ*_*p*_ Represents the autoregressive coefficient, *θ*_1_, ⋯, *θ*_*q*_ Represents the moving average coefficient, {*ε*_*t*_} Represents the white noise sequence.

The ARMA model can be transformed into the ARIMA model by weakening the trend factors through the differencing. An ARIMA model can be transformed into a SARIMA model by dealing with the seasonal factors of time series [35]. We can flexibly use the ARIMA model according to the characteristics of time series.

The differencing can further stabilize the unstable time series to obtain a stable time series [21], series {*Y*_*t*_} can become a stationary sequence series {*X*_*t*_} after *d* times of differencing, which is described by the equation:

$$X_{t}=\nabla^{d}y_{t}={\left(1-B\right)}^{d}y_{t}$$ (9)

In the Eq (9), ∇ = 1 − *B* represents the differencing operator, and B represents the lag operator.

By combining the differencing and ARMA(p,q) model, the ARIMA(p,d,q) model can be obtained, where *p* represents the autoregressive order, *d* is the order of differencing *q* represents the moving average order.

#### 3.6.1 Model identification

The differencing equation is as follows:

$${X_{t}}^{\prime }=X_{t}-X_{t-1}$$ (10)

**Fig 17:**
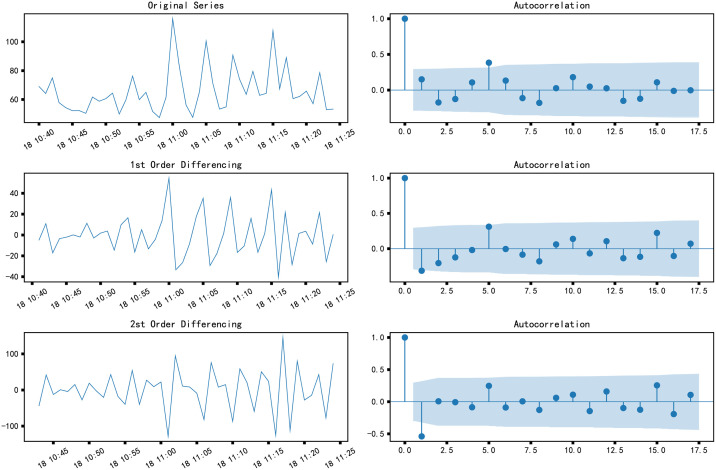
Autocorrelation diagram of differential sequence.

ACF can also subjectively judge the stationarity of time series [35]. As can be seen from the ACF diagram on the correct (Fig 17), after the second differencing, the lag value soon enters the negative value area and exceeds the confidence area, which indicates that the time series may have been over differencing. Therefore, we temporarily set the order of the differencing to 1.

The stationarity of the time series can be judged by ADF [37]. If the p-value of the unit root test statistic is less than the significance level (0.05), reject the original hypothesis and infer that the time series is stationary.

ADF test is carried out on the time series. Tables 3 and 4 are the ADF test results. The results show that after one differencing, the significance level of the sample is higher than 0.05, and the time series has been stable.

**Table 3:** Original sequence of ADF test results.

		T-Statistic
Augmented Dickey-Fuller test statistic		-1.810635
Test critical values	1% level	-3.574589
	5% level	-2.923954
	10% level	-2.600039

**Table 4:** Results of 1st order differencing.

		T-Statistic
Augmented Dickey-Fuller test statistic		-8.012107
Test critical values	1% level	-3.574589
	5% level	-2.923954
	10% level	-2.6000391

The stationary time series is divided into training and test sets. The data set consists of 61 mean observations per minute, 80% of which are used to build the model (49 observations), called the training set. The interval of the training set is from 10:40 to 11:28, and the remaining 20% (12 observations) are used to verify the prediction of the model, called the test set. The test set interval is from 11:29 to 11:40.

#### 3.6.2 Parameter estimation

*(1) ACF and PACF*. ACF function equation is as follows:

$$\text{ACF}(k)=\rho_{k}=\frac{Cov(y_{t},y_{t-k})}{Var(y_{t})}$$ (11)

In the function, *k* represents the lag order. ACF can measure the correlation between *y*_*t*_, *y*_*t*_ *y*_*t*−*k*_. PACF Can eliminate the interference of *k* − 1 random variables between *x*(*t*) and *x*(*t* − *k*).

In general, parameters p and q can be determined by significant lag order in PACF and ACF graphs [47], but parameters identified based on ACF and PACF can not be quantitatively analyzed.

*(2) AIC and BIC*. It is also a standard method to use AIC and BIC to select models. Usually, models with minimum Akaike information Criterion or minimum Bayesian information Criterion are selected [39]. The equations of AIC and BIC are as follows:

AIC=2k−2ln(L) (12)

BIC=kln(n)−2ln(L) (13)

*k* in the equation represents the number of unknowns in the model, L represents the maximum likelihood function of the model, and n represents the number of samples.

**Fig 18:**
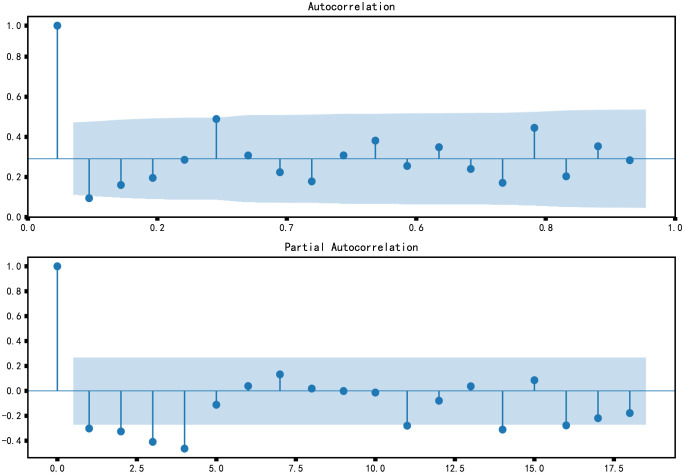
ACF and PACF.

Autocorrelation graph and partial autocorrelation graph are shown in Fig 18, which shows that the order p of autoregression is at order 0, p can also be at order 4, and the order of moving average is at order 0 or 1.

**Fig 19:**
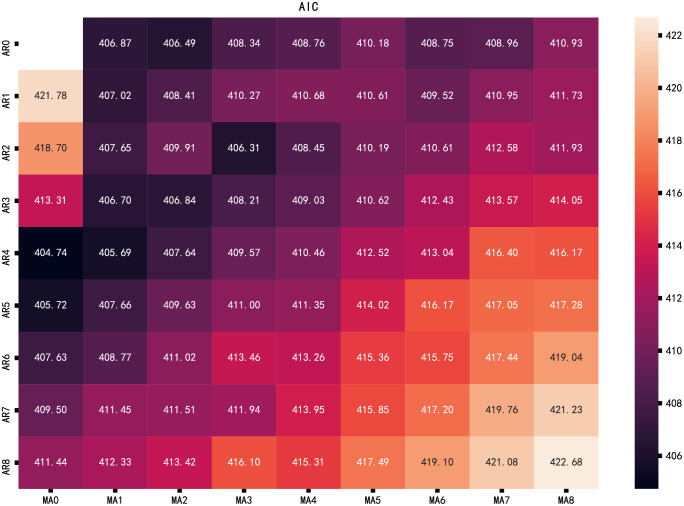
AIC thermal diagram.

**Fig 20:**
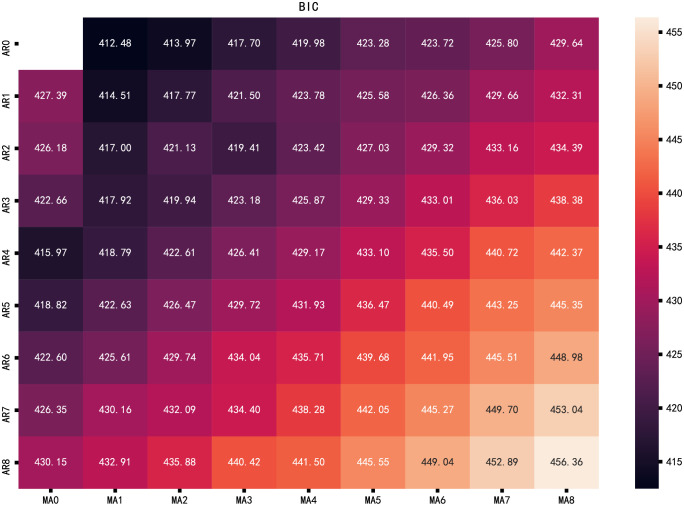
BIC thermal diagram.

AIC and BIC were used to evaluate the parameters. The parameters were selected in the range 0 to 8, the values of AIC and BIC were obtained by cyclic calculation. The calculation results were presented in the thermal diagram. The smaller the AIC (Fig 19) or BIC (Fig 20), the darker the color in the thermal diagram. It can be seen that the best parameter combination (p, q) is (4,0) and (0,1), AIC value is 404.74 and 406.87, BIC value is 415.97 and 412.48, respectively. These combinations are consistent with subjective judgment based on autocorrelation and partial autocorrelation graphs.

#### 3.6.3 Diagnostics

There are many combinations of ARIMA model parameters. In order to evaluate the model established by different parameters, a diagnosis of the model is required. Using root mean Square Error (RMSE), the mean absolute percentage error (MAPE) can measure the accuracy of the fitting model [48], Then, the steadiness of the residual is checked, and the normality of the residual is evaluated [47], Ljung-Box is used to test whether the residual of the model belongs to white noise [40], to evaluate the goodness of fit of the model. The evaluation criteria for the residual of the model belonging to the white noise sequence are as follows: The assumption that the residual is at the confidence level of 95.0% or higher cannot be rejected, meaning that the p-value is greater than or equal to 0.05 [48].

We used RMSE and MAPE to evaluate the error of prediction results [49]. The equation of RMSE and MAPE is as follows:

$$RMSE=\sqrt{\frac{1}{m}\sum_{i=1}^{m}{\left(y_{i}-{\hat{y}}_{i}\right)}^{2}}$$ (14)

$$\text{MAPE}=\frac{100\%}{m}\sum_{i=1}^{m}\left|{\hat{y}}_{i}-y_{i}\right|$$ (15)

**Fig 21:**
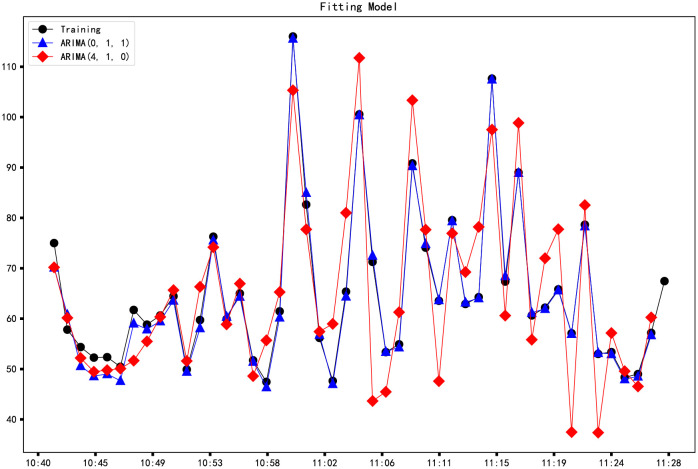
ARIMA (0,1,0) model and ARIMA (4,1,0) model.

**Fig 22:**
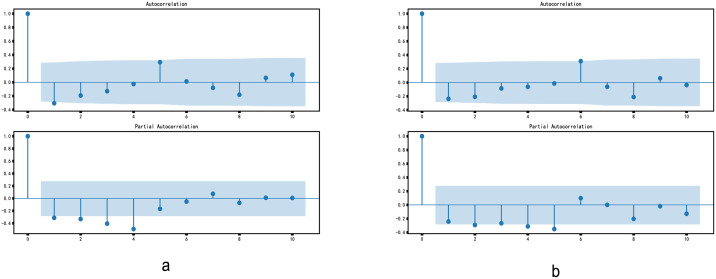
Residual autocorrelation diagram and partial autocorrelation diagram of ARIMA (0,1,1) (a) and ARIMA (4,1,0) (b).

From the previous section, we know that the best parameter combination (p, q) of the current sample is (4,0) and (0,1), and AIC and BIC are very small. ARIMA (4,1,0) and ARIMA (0,1,1) are used for model fitting, and the fitting results are shown in (Fig 21). Finally, the residual error is analyzed. According to the residuals of ARIMA (4,1,0) and ARIMA (0,1,1), the autocorrelation diagram and partial autocorrelation diagram are plotted in (Fig 22). It can be seen that the residuals of ARIMA (4, 1, 0) and ARIMA (0, 1, 1) do not show an obvious correlation. It can be seen from Table 5 that the MAPE and RMSE of the ARIMA (0,1,1) model are more petite than ARIMA (4,1,0).

**Table 5:** Characteristics of the best Arima fitting model for PM2.5 concentration in the training set.

Model	RMSE	MAPE	AIC(BIC)
ARIMA (0,1,1)	1.527	1.7%	406.87(412.48)
ARIMA (4,1,0)	8.919	10.5%	404.74(415.97)

**Fig 23:**
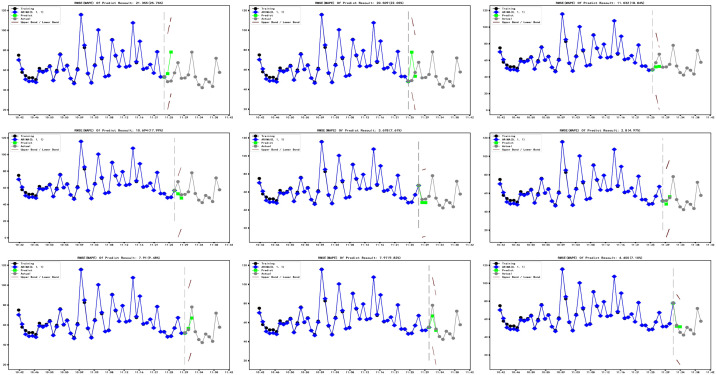
ARIMA (0,1,1) model cross-validation.

**Fig 24:**
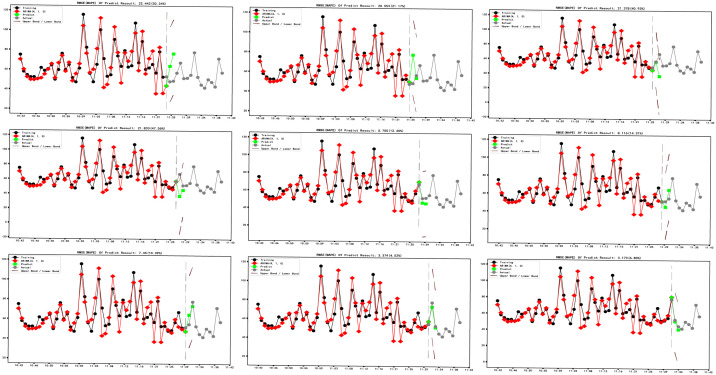
ARIMA (4,1,0) model cross-validation.

In order to further evaluate the performance of the model, we conduct cross-validation (Figs 23 and 24), input data as time advances, and predict forward for two minutes. The prediction results are compared with the test set.

As shown in (Fig 24), with the continuous input of data, the prediction accuracy of the ARIMA (0,1,1) model and ARIM (4,1,0) model is continuously improved. The average MAPE of the ARIMA (0,1,1) model in cross-validation is 13.74%, and the lowest MAPE can reach 4.97%. The average MAPE of the ARIMA (4,1,0) model in cross-validation is 20.92%, and the lowest MAPE can reach 4.52%.

From the results, the ARIMA (0,1,1) model is the best. However, after the Ljung-Box test, we found that the test value (p-value) of the residual of the ARIMA (0,1,1) model is 0.029 (less than 0.05), and the residual of ARIMA (0,1,1) model is not white noise. The test value (p-value) of the residual of ARIMA (4,1,0) model is 0.091 (greater than 0.05).

The results show that ARIMA (0,1,1) model is overfitting, and the sample data has not been fully utilized. Therefore, ARIMA (4,1,0) model is selected.

**Fig 25:**
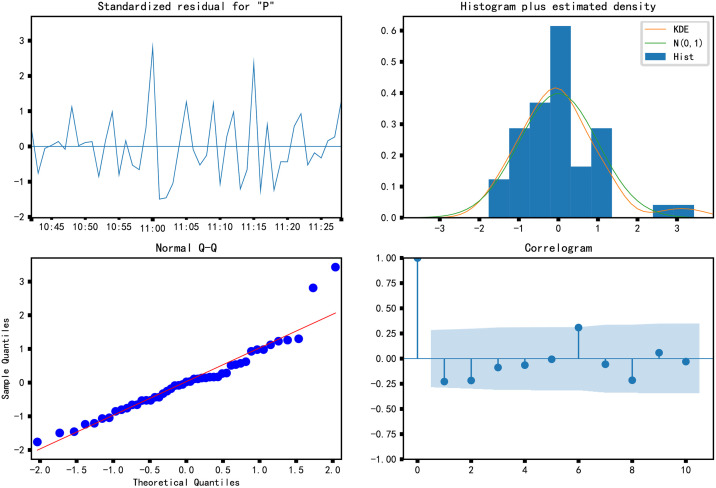
Residual analysis.

Further analysis of the residuals of the ARIMA (4,1,0) model (Fig 25), according to the upper left corner of the graph, the residuals have no evident seasonality, similar to the white noise sequence and the residual autocorrelation graph in the lower right corner confirms this point. The autocorrelation coefficients are between the confidence intervals, and the residuals pass the independence test. The Normal Distribution diagram in the upper right corner shows that the residuals are normally distributed, and the QQ diagram in the lower-left corner also shows that the distribution of the residuals follows the standard normal distribution. Therefore, the ARIMA (4,1,0) model is the best model for the current time series.

## 4. Discuss

The comparison of prediction effects between the DES model and ARIMA model is shown in the following Table 6:

**Table 6:** Characteristic evaluation of DES model and ARIMA (4,1,0,).

Model	Fitting model RMSE	Fitting model MAPE	CV prediction average RMSE	CV prediction average MAPE
DES	4.03	5.72%	8.34	12.9%
ARIMA (4,1,0)	8.919	10.5%	12.675	20.92%

Under the currently selected time-series samples, the two models have the following performances:

1. The modeling speed of the DES model is faster than that of the ARIMA model.
2. It can be seen from the equation of the DES model that the prediction range of the DES model is one step ahead.
3. The prediction range of the ARIMA model is adjustable
4. From the cross-validation diagram (Figs 16 and 24), it can be seen that the accuracy of the DES model does not change significantly with the input of data. The difference is that the accuracy of the prediction model is significantly improved with the input of data. ARIMA model relies more on PM2.5 changes in sample length.
5. The ARIMA model has strong expansibility and can also deal with seasonal factors data.
6. Considering the randomness of PM2.5 dust on the opencast coal mine road, the ARIMA model’s prediction result is acceptable.

Due to the significant fluctuation of PM2.5 dust concentration on the road of the opencast coal mine, the short-term prediction is more in line with the actual production requirements. Therefore, the 2-minute prediction range meets the basic requirements of PM2.5 concentration early warning on the road of the opencast coal mines. Moreover, the results of cross-validation prove the excellent generalization ability of the ARIMA model. Therefore, to improve the feasibility and expansibility of the prediction model in the prediction system, the ARIMA model is more suitable for the short-term prediction of PM2.5 concentration in opencast coal mine roads.

## 5. Prediction system

**Fig 26:**
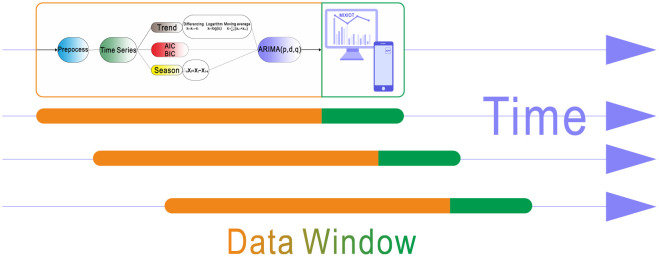
Prediction process.

As shown in Fig 26, the monitoring system’s data continues to enter the prediction system, and the prediction system continues to predict through the mobile data window.

The original data becomes the standard time series in the prediction system after preprocessing. The time series is stabilized by eliminating the trend and seasonal components of the time series. The criterion of time series stabilization is whether the p-value in the ADF test is less than 0.05. When using AIC and BIC to select parameters, we may encounter a variety of parameter combinations, but MAPE and RMSE can evaluate the fitting accuracy, and Ljun-box can be used to test the best model. When the data enters the above process, the best prediction model in the current data window can be selected, and the prediction range of the model is set to two minutes.

The data continue to enter the model over time, and the model continues to predict forward. After each prediction, the current model’s performance is judged by comparing the MAPE and RMSE between the historical data and the prediction results. There are quantitative evaluation indexes for stability evaluation, parameter selection, and the best model test. Therefore, the program will re-establish the prediction model and predict the next data window when the model is no longer applicable.

The functions of the whole monitoring and prediction system include data acquisition, data preprocessing, model construction, prediction, early warning, and data visualization.

## 6. Conclusion

1. At present, the environmental monitoring ability of opencast coal mines is low, the amount of data collection is small, and the data acquisition is not real-time. Moreover, building multi-sensor monitoring nodes is very complex, which means that subsequent expansion and upgrading are difficult. In this study, the APRUS adapter is used to simplify the construction process of a multi-sensor monitoring node. We have successfully constructed a PM2.5 dust monitoring node on an opencast coal mine road. At the same time, we have also realized the real-time acquisition of PM2.5 dust information on the road of an opencast coal mine, visual monitoring, and overrun alarm. The user-defined visualization function of the Internet of things platform facilitates managers’ learning without programming experience. This research provides a complete design process, prototype, and guidance for constructing an opencast coal mine monitoring system.
2. In the cross-validation results of time series, the average MAPE of the ARIMA model and DES model are 20.92% and 12.9%, respectively, and the lowest can reach less than 5%, which proves that the DES model and ARIMA model have good feasibility and accuracy in the short-term prediction of PM2.5. The DES model is simple to build, but it can also obtain high short-term prediction accuracy. In the case of only pursuing the prediction accuracy, using the DES model is a good alternative. On the other hand, in the process of cross-validation, the prediction accuracy of the ARIMA model will improve with the input of data. The ARIMA model is sensitive to the real-time change of PM2.5, so the ARIMA model is suitable for PM2.5 real-time monitoring data with significant fluctuation. Because the prediction range of the ARIMA model is adjustable and the use of the ARIMA model is flexible, the ARIMA model is more suitable for the PM2.5 prediction system of opencast coal mine road.

In the future, the open-source Internet of things platform needs to be linked with dust reduction equipment so that we can take dust reduction measures before the dust concentration exceeds the threshold to protect the respiratory health of workers in opencast coal mines.
